# Evaluating cancer research impact: lessons and examples from existing reviews on approaches to research impact assessment

**DOI:** 10.1186/s12961-020-00658-x

**Published:** 2021-03-11

**Authors:** Catherine R. Hanna, Kathleen A. Boyd, Robert J. Jones

**Affiliations:** 1 CRUK Clinical Trials Unit, Institute of Cancer Sciences, University of Glasgow, Glasgow, United Kingdom; 2 Health Economics and Health Technology Assessment, Institute of Health and Wellbeing, University of Glasgow, Glasgow, United Kingdom

**Keywords:** Impact, Research, Cancer, Oncology, Trials, Evaluation, Methods

## Abstract

**Background:**

Performing cancer research relies on substantial financial investment, and contributions in time and effort from patients. It is therefore important that this research has real life impacts which are properly evaluated. The optimal approach to cancer research impact evaluation is not clear. The aim of this study was to undertake a systematic review of review articles that describe approaches to impact assessment, and to identify examples of cancer research impact evaluation within these reviews.

**Methods:**

In total, 11 publication databases and the grey literature were searched to identify review articles addressing the topic of approaches to research impact assessment. Information was extracted on methods for data collection and analysis, impact categories and frameworks used for the purposes of evaluation. Empirical examples of impact assessments of cancer research were identified from these literature reviews. Approaches used in these examples were appraised, with a reflection on which methods would be suited to cancer research  impact evaluation going forward.

**Results:**

In total, 40 literature reviews were identified. Important methods to collect and analyse data for impact assessments were surveys, interviews and documentary analysis. Key categories of impact spanning the reviews were summarised, and a list of frameworks commonly used for impact assessment was generated. The Payback Framework was most often described. Fourteen examples of impact evaluation for cancer research were identified. They ranged from those assessing the impact of a national, charity-funded portfolio of cancer research to the clinical practice impact of a single trial. A set of recommendations for approaching cancer research impact assessment was generated.

**Conclusions:**

Impact evaluation can demonstrate if and why conducting cancer research  is worthwhile. Using a mixed methods, multi-category assessment organised within a framework, will provide a robust evaluation, but the ability to perform this type of assessment may be constrained by time and resources. Whichever approach is used, easily measured, but inappropriate metrics should be avoided. Going forward, dissemination of the results of cancer research impact assessments will allow the cancer research community to learn how to conduct these evaluations.

**Supplementary information:**

**Supplementary information** accompanies this paper at 10.1186/s12961-020-00658-x.

## Background

Cancer research attracts substantial public funding globally. For example, the National Cancer Institute (NCI) in the United States of America (USA) had a 2020 budget of over $6 billion United States (US) dollars. In addition to public funds, there is also huge monetary investment from private pharmaceutical companies, as well as altruistic investment of time and effort to participate in cancer research from patients and their families. In the United Kingdom (UK), over 25,000 patients were recruited to cancer trials funded by one charity (Cancer Research UK (CRUK)) alone in 2018 [1]. The need to conduct research within the field of oncology is an ongoing priority because cancer is highly prevalent, with up to one in two people now having a diagnosis of cancer in their lifetime [2, 3], and despite current treatments, mortality and morbidity from cancer are still high [2].

In the current era of increasing austerity, there is a desire to ensure that the money and effort to conduct any type of research delivers tangible downstream benefits for society with minimal waste [4–6]. These wider, real-life benefits from research are often referred to as research impact. Given the significant resources required to conduct cancer research in particular, it is reasonable to question if this investment is leading to the longer-term benefits expected, and to query the opportunity cost of not spending the same money directly within other public sectors such as health and social care, the environment or education.

The interest in evaluating research impact has been rising, partly driven by the actions of national bodies and governments. For example, in 2014, the UK government allocated its £2 billion annual research funding to higher education institutions, in part based on an assessment of the impact of research performed by each institution in an assessment exercise known as the Research Excellence Framework (REF). The proportion of funding dependent on impact assessment will increase from 20% in 2014, to 25% in 2021[7].

Despite the clear rationale and contemporary interest in research impact evaluation, assessing the impact of research comes with challenges. First, there is no single definition of what research impact encompasses, with potential differences in the evaluation approach depending on the definition. Second, despite the recent surge of interest, knowledge of how best to perform assessments and the infrastructure for, and experience in doing so, are lagging [6, 8, 9]. For the purposes of this review, the definition of research impact given by the UK Research Councils is used (see Additional file 1 for full definition). This definition was chosen because it takes a broad perspective, which includes academic, economic and societal views of research impact [10].

There is a lack of clarity on how to perform research impact evaluation, and this extends to cancer research. Although there is substantial interest from cancer funders and researchers [11], this interest is not accompanied by instruction or reflection on which approaches would be suited to assessing the impact of cancer research specifically. In a survey of Australian cancer researchers, respondents indicated that they felt a responsibility to deliver impactful research, but that evaluating and communicating this impact to stakeholders was difficult. Respondents also suggested that the types of impact expected from research, and the approaches used, should be discipline specific [12]. Being cognisant of the discipline specific nature of impact assessment, and understanding the uniqueness of cancer research in approaching such evaluations, underpins the rationale for this study.

The aim of this study was to explore approaches to research impact assessment, identify those approaches that have been used previously for cancer research, and to use this information to make recommendations for future evaluations. For the purposes of this study, cancer research included both basic science and applied research, research into any malignant disease, concerning paediatric or adult cancer, and studies spanning nursing, medical, public health elements of cancer research.

The study objectives were to:

i. Identify existing literature reviews that report approaches to research impact assessment and summarise these approaches.
ii. Use these literature reviews to identify examples of cancer research impact evaluations, describe the approaches to evaluation used within these studies, and compare them to those described in the broader literature.

This approach was taken because of the anticipated challenge of conducting a primary review of empirical examples of cancer research impact evaluation, and to allow a critique of empirical studies in the context of lessons learnt from the wider literature. A primary review would have been difficult because examples of cancer research impact evaluation, for example, the assessment of research impact on clinical guidelines [13], or clinical practice [14–16], are often not categorised in publication databases under the umbrella term of research impact. Reasons for this are the lack of medical subject heading (MeSH) search term relating to research impact assessment and the differing definitions for research impact. In addition, many authors do not recognise their evaluations as sitting within the discipline of research impact assessment, which is a novel and emerging field of study.

## Methods

### General approach

A systematic search of the literature was performed to identify existing reviews of approaches to assess the impact of research. No restrictions were placed on the discipline, field, or scope (national/global) of research for this part of the study. In the second part of this study, the reference lists of the literature reviews identified were searched to find empirical examples of the evaluation of the impact of cancer research specifically.

### Data sources and searches

For the first part of the study, 11 publication databases and the grey literature from January 1998 to May 2019 were searched. The electronic databases were Medline, Embase, Health Management and Policy Database, Education Resources Information Centre, Cochrane, Cumulative Index of Nursing and Allied Health Literature, Applied Social Sciences Index and Abstract, Social Services Abstracts, Sociological Abstracts, Health Business Elite and Emerald. The search strategy specified that article titles must contain the word “impact”, as well as a second term indicating that the article described the evaluation of impact, such as “model” or “measurement” or “method”. Additional file 1 provides a full list of search terms. The grey literature was searched using a proforma. Keywords were inserted into the search function of websites listed on the proforma and the first 50 results were screened. Title searches were performed by either a specialist librarian or the primary researcher (Dr. C Hanna). All further screening of records was performed by the primary researcher.

Following an initial title screen, 800 abstracts were reviewed and 140 selected for full review. Articles were kept for final inclusion in the study by assessing each article against specific inclusion criteria (Additional file 1). There was no assessment of the quality of the included reviews other than to describe the search strategy used. If two articles drew primarily on the same review but contributed a different critique of the literature or methods to evaluate impact, both were kept. If a review article was part of a grey literature report, for example a thesis, but was also later published in a journal, the journal article only was kept. Out of 140 articles read in full, 27 met the inclusion criteria and a further 13 relevant articles were found through reference list searching from the included reviews [17].

**Fig. 1:**
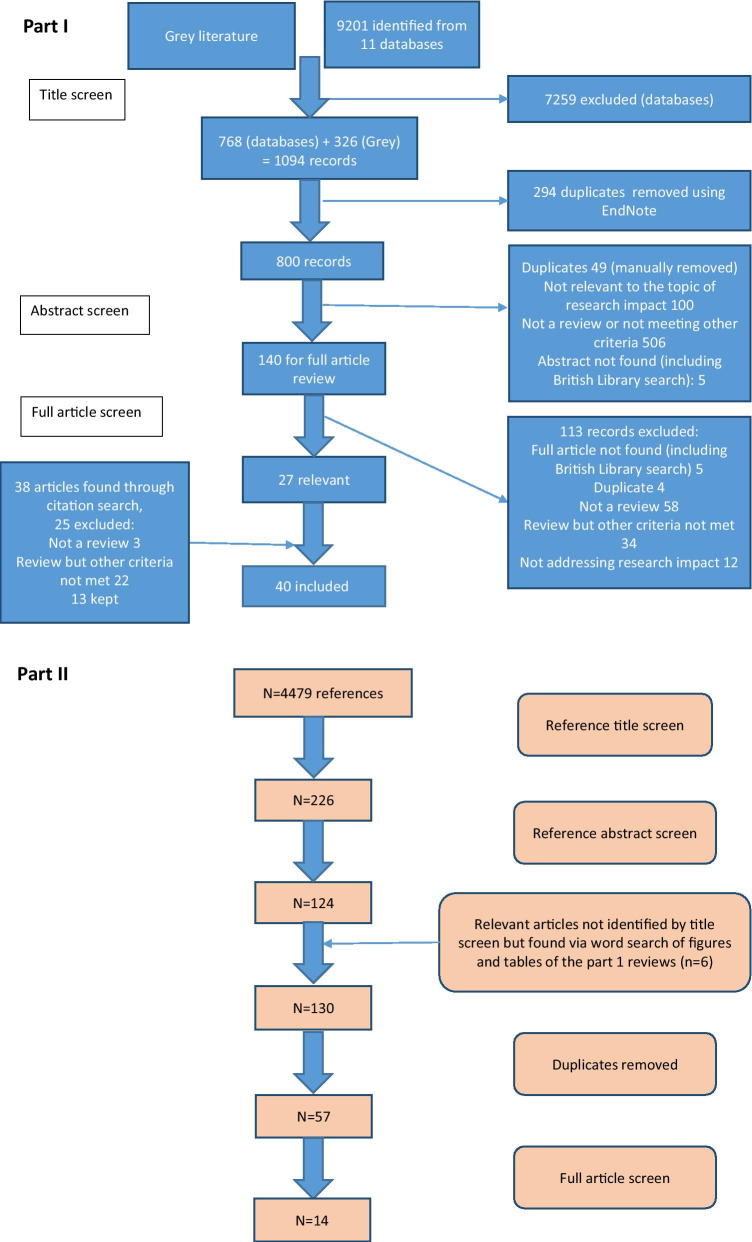
Search strategies for this study

For the second part of the study, the reference lists from the literature reviews were manually screened [17] (*n* = 4479 titles) by the primary researcher to identify empirical examples of assessment of the impact of cancer research. Summary tables and diagrams from the reviews were also searched using the words “cancer” and “oncology” to identify relevant articles that may have been missed by reference list searching. After removal of duplicates, 57 full articles were read and assessed against inclusion criteria (Additional file 1). Figure 1 shows the search strategy for both parts of the study according to the guidelines for preferred reporting items for systematic reviews and meta-analysis (PRISMA) [18].

### Data extraction and analysis

A data extraction form produced in Microsoft^®^ Word 2016 was used to collect details for each literature review. This included year of publication, location of primary author, research discipline, aims of the review as described by the authors and the search strategy (if any) used. Information on approaches to impact assessment was extracted under three specific themes which had been identified from a prior scoping review as important factors when planning and conducting research impact evaluation. These themes were: (i) categorisation of impact into different types depending on who or what is affected by the research (the individuals, institutions, or parts of society, the environment), and how they are affected (for example health, monetary gain, sustainability) (ii) methods of data collection and analysis for the purposes of evaluation, and (iii) frameworks to organise and communicate research impact. There was space to document any other key findings the researcher deemed important. After data extraction, lists of commonly described categories, methods of data collection and analysis, and frameworks were compiled. These lists were tabulated or presented graphically and narrative analysis was used to describe and discuss the approaches listed.

For the second part of the study, a separate data extraction form produced in Microsoft^®^ Excel 2016 was used. Basic information on each study was collected, such as year of publication, location of primary authors, research discipline, aims of evaluation as described by the authors and research type under assessment. Data was also extracted from these empirical examples using the same three themes as outlined above, and the approaches used in these studies were compared to those identified from the literature reviews. Finally, a set of recommendations for future evaluations of cancer research impact were developed by identifying the strengths of the empirical examples and using the lists generated from the first part of the study to identify improvements that could be made.

## Results

### Part one: Identification and analysis of literature reviews describing approaches to research impact assessment

#### Characteristics of included literature reviews

Forty literature reviews met the pre-specified inclusion criteria and the characteristics of each review are outlined in Table 1. A large proportion (20/40; 50%) were written by primary authors based in the UK, followed by the USA (5/40; 13%) and Australia (5/40; 13%), with the remainder from Germany (3/40; 8%), Italy (3/40; 8%), the Netherlands (1/40; 3%), Canada (1/40; 3%), France (1/40; 3%) and Iran (1/40; 3%). All reviews were published since 2003, despite the search strategy dating from 1998. Raftery et al. 2016 [19] was an update to Hanney et al. 2007 [20] and both were reviews of studies assessing research impact relevant to a programme of health technology assessment research. The narrative review article by Greenhalgh et al. [21] was based on the same search strategy used by Raftery et al. [19].

**Table 1:** Summary of literature reviews

Reference ID number	Author	Year	Location	Main aims	Search strategy (1 = yes, 0 = no)	Search Methods	Keywords list (1 = yes, 0 = no)	PRISMA (1 = yes, 0 = no)	Timeline and language	Discipline of research/perspective	Methods of data collection (1 = included, 0 = not included)	Categories (1 = included, 0 = not included)	Frameworks (1 = included, 0 = not included)	Table or diagram of empirical impact assessments (1 = included, 0 = not included)
1	Hanney et al.	2003	UK	To review how health research is used in policy-making and the approaches to assess the policy impact of research	0	NA	0	0	Unknown	Healthcare	1	0	1	1
2	Buxton et al.	2004	UK	To identify key studies that have estimated the economic value of the impact of health research to society	1	Databases (11 in total) and grey literature	1	0	Unknown/English only	Healthcare	1	0	0	1
3	Coryn et al.	2007	USA	To describe, classify, and comparatively evaluate national models and mechanisms used to evaluate research in 16 countries	0	NA	0	0	Unknown	General	0	0	1	0
4	Hanney et al. (Chapter 2)	2007	UK	To review the literature describing the evaluation of the impact of programmes of health research to inform an evaluation of the impact of the first 10 years of the NHS HTA programme from its inception in 1993 to June 2003	1	Database (13 in total), citation analysis, expert consultation, advisory group consultation	1	0	1990–2005/unknown	Healthcare	1	0	1	1
5	Brutsher et al. (Part 1 of report)	2008	UK	To present and discuss five key elements of research evaluation	0	NA	0	0	Unknown	General	1	1	1	0
6	Buxton et al. (Chapter 2)	2008	UK	To review current practices of assessing the economic benefits of health research	0	NA	0	0	Unknown	Medical research	1	0	0	0
7	Boaz et al.	2009	UK	A literature review to explore methods for evaluating the impact of research on policy outcomes that might be appropriate to the Sustainable Waste and Resource Management and the Sustainable Consumption and Production research programmes	1	Databases (10 in total), web searches of 30 organisation websites, citation tracking, expert contacts	1	0	1987–2007/English only	Cross-sector but written for the Environment, food and rural affairs department of the UK government	1	0	1	0
8	Marjanovic et al.	2009	UK	To give a historical overview of landmark studies in the research evaluation field. To use this historical account to reflect on the methodological developments and recurrent themes in research evaluation studies	0	NA	0	0	Unknown	Biomedical and health research	1	0	1	0
9	Yazdizadeh et al.	2010	Iran	A systematic review to identify the methods used to assess the economic impact of healthcare research, and the outcomes	1	Databases (8 in total), 21 relevant websites	1	0	Unknown	Healthcare	1	0	1	0
10	Banzi et al.	2011	Italy	To identify the most common approaches to research impact assessment, categories of impact and their respective indicators	1	Databases (2 in total), research funding and charity's foundations websites cited in the studies, citation screening	0	0	1990–2009/English, French, Spanish, Italian	General	1	1	1	0
11	Hanney et al.	2011	UK	To review studies that have attempted to assess economic impacts from health research in the field of nursing health research	1	Databases (2 in total), reviews of retrospective studies already known to the authors	0	0	Unknown	Healthcare	1	0	0	1
12	Patel et al.	2011	UK	A systematic review to identify the indicators that have been used to measure healthcare research performance	1	Databases (4 in total), citation screening	1	1	1950–2010/No restrictions	Healthcare	1	0	1	0
13	Ruscio et al.	2012	USA	To evaluate 22 scholarly impact metrics, including conventional measures, the h index, and many variations on the h theme	0	NA	0	0	Unknown	General	1	0	0	1
14	Bornmann et al.	2013	Germany	To present existing research on and practices employed in the assessment of societal impact in the form of a literature survey	1	Databases (2 mentioned, total not clear), internet search engines, citation screening	0	0	Unknown	General	1	0	1	1
15	Guthrie et al.	2013	UK	To identify and review frameworks in use for research evaluation, to identify the research evaluation tools applied to those frameworks to provide a guide to developing a research evaluation framework that can be used in a range of circumstances	0	NA	0	0	Unknown	General	1	0	1	0
16	Smith et al.	2013	UK	To review the methods of assessing research impact that are relevant to academic promotion	1	Databases (5 in total), internet search engine, citation screening	1	0	Unknown	General	1	0	1	0
17	Carpenter et al.	2014	USA	To provide a broad overview of widely available measures of academic productivity and impact using publication data and to highlight the uses of these metrics for various purposes	0	NA	0	0	Unknown	Science	1	0	0	0
18	Penfield et al.	2014	UK	To explore what is understood by the term research impact, and to provide a comprehensive overview of the literature to understand which methods and frameworks of impact assessment could be used for UK impact assessment	0	NA	0	0	Unknown	General	1	0	1	0
19	Milat et al.	2015	Australia	To synthesize evidence that describes processes and conceptual models for assessing policy and practice impacts of public health research	1	Databases (6 in total)	1	1	1990–2013/English only	Healthcare	1	0	1	0
20	Moed et al.	2015	The Netherlands	To provide a broad overview of the wide array of metrics to assess research impact currently in use in academia and research	0	NA	0	0	Unknown	General	1	1	1	0
21	Pollitt et al. (Appendix 2)	2015	UK	To identify a wide range of potential impacts of research, investigate different ways of classifying impacts, produce a possible long list of types of impact and domains for impact	1	Search "Largely covered grey literature as well as some academic literature and focused initially on a limited set of key sources known to the project team and advisory board."	0	0	Unknown	General	0	1	1	0
22	Thonon et al.	2015	France	To identify and critique indicators of impact that could be used to measure the output and outcome of medical research	1	Databases Snowballing	1	1	Unknown/French or English	Biomedical research	1	1	0	0
23	Wouters et al.	2015	UK	A literature review of academic research looking a range of impact indicators that may be useful in research evaluations, including the next Research Excellence Framework (REF)	0	NA	0	0	Unknown	General	1	0	0	0
24	Agarwal et al.	2016	USA	To provide a broad overview of the wide array of evaluation metrics currently in use in academia and research	0	NA	0	0	Unknown	General	1	0	0	0
25	Chikoore	2016	UK	To explore the meaning of research impact, the issues regarding how it can be evaluated, the methods used in evaluating this research impact and identifying the challenges of these evaluations	0	NA	0	0	Unknown	General	1	0	1	0
26	Raftery et al.	2016	UK	To review published research studies on tools and approaches to assessing the impact of programmes of health research and, specifically, to update the previous 2007 systematic review funded by the HTA programme	1	Databases (8 in total), hand searching selected journals, citation screening, literature known to the research team already (snowballing), bibliographic searches of other reviews and references identified in relevant literature	1	1	2005–2014/Unknown	Healthcare	1	1	1	1
27	Greenhalgh et al.	2016	UK	To review the strengths and limitations of six established approaches of measuring both the outcomes of research and the processes and activities through which this is achieved	0	NA	0	0	Search strategy based on Raftery et al.	General	1	0	1	1
28	Wimmer et al.	2016	USA	To review both traditional and more novel impact evaluation tools, the impact metrics they calculate, and why the tools are particularly relevant to the field of nursing	0	NA	0	0	Unknown	General	1	0	0	0
29	Bornmann et al.	2017	Germany	To review how impact is measured within science and beyond, the effects that impact measurements have on the science system and which problems are associated with impact measurement	1	Databases (3 in total) and other literature reviews	1	0	Unknown	Science	1	0	0	0
30	Cruz Rivera et al.	2017	UK	To identify the existing methodological frameworks used to measure healthcare research impact and to summarise the common themes and metrics in an impact matrix	1	Databases (4 in total), internet search engines including images, communication with experts	1	1	Not specified (Appendix 1 containing full search strategy also checked)	Healthcare	0	1	1	0
31	Deeming et al.	2017	Australia	To list the stated objectives for research impact assessment frameworks, to identify existing frameworks and to evaluate whether the identified frameworks possessed the capabilities necessary to address the specified objectives	1	Database (Scopus) and grey literature	1	1	2005–2015/English only	Healthcare/Medical	1	0	1	0
32	Peter et al.	2017	Canada	To identify the approaches that have been used to understand the impacts of health research, to identify ways that research impacts have been defined and measured and to provide recommendations for occupational science	1	Traditional databases, author search from the included articles, assessment tools that were mentioned in the included reviews used as keywords to search traditional databases, reverse citation analysis and a forward citation search in the Scopus database	1	1	No restrictions	Occupational science	1	0	1	0
33	Reale et al.	2017	Italy	To understand how the impact assessment methods used in social sciences and humanities and how far these approaches attempt to apply methods and instruments that take into account the distinctive features of this discipline	1	Journal articles/databases Books Reports Working papers CORDIC database EU FP17 Flash-it project Web searchers Guidelines for applicants and evaluators Grey literature Snowballing	0	0	2006–2012. Mostly English	Social sciences and humanities	1	1	1	0
34	Newson et al.	2018	Australia	To review the extent and nature of studies measuring the impacts of health research on policy and compare forward and backward tracing approaches for assessment	1	Electronic databases (5) References of included studies	1	1	1995–2016. English only	General	1	0	1	1
35	Pedrini et al.	2018	Italy	To analyse the approaches to the assessment of healthcare research's social impact with a focus on different stakeholders	1	Databases (3 in total)	1	0	2000–2016/Unknown	Healthcare	1	0	0	1
36	Weisshuhn et al.	2018	Germany	To conduct a literature review to analyse how impacts of agricultural research are assessed	1	Databases	0	0	2008–2016/Unknown	Agricultural	1	1	0	0
37	Williams et al.	2018	UK	To provide a systematic review of the evolution of research impact assessment approaches in Australia and the UK	1	Public policy documents Newspaper commentary Academic literature	0	0	Unknown	General	0	0	1	0
38	Braithwaite et al.	2019	Australia	To identify what is known about methods for assessing researchers’ achievements for the purposes of producing a new assessment model	1	Databases (all Web of Science databases)	1	1	2007–2017. English only	General	1	0	0	0
39	Gomes et al	.2019	UK	To review empirical impact evaluations to understand the impact generated by publicly and charity-funded health research in the UK	1	Databases Citation tracking Reference searching of including articles Hand searching of specific journals	1	1	2006–2017. English only	Health research	1	0	1	1
40	Heyeres et al	2019	Australia	To perform a systematic review of studies that used a case study approach to assess research impact	1	Databases (11) Reference list of impact case studies identified	1	1	2000–2018. English only	General	1	0	1	1

Approximately half of the reviews (19/40; 48%) described approaches to evaluate research impact without focusing on a specific discipline and nearly the same amount (16/40; 40%) focused on evaluating the impact of health or biomedical research. Two reviews looked at approaches to impact evaluation for environmental research and one focused on social sciences and humanities research. Finally, two reviews provided a critique of impact evaluation methods used by different countries at a national level [22, 23]. None of these reviews focused specifically on cancer research.

Twenty-five reviews (25/40; 63%) specified search criteria and 11 of these included a PRISMA diagram. The articles that did not outline a search strategy were often expert reviews of the approaches to impact assessment methods and the authors stated they had chosen the articles included based on their prior knowledge of the topic. Most reviews were found by searching traditional publication databases, however seven (7/40; 18%) were found from the grey literature. These included four reports written by an independent, not-for-profit research institution (Research and Development (RAND) Europe) [23–26], one literature review which was part of a Doctor of Philosophy (Ph.D) thesis [27], a literature review informing a quantitative study [28] and a review that provided background information for a report to the UK government on the best use of impact metrics [29].

#### Key findings from the reviews: approaches to research impact evaluation

**Fig. 2:**
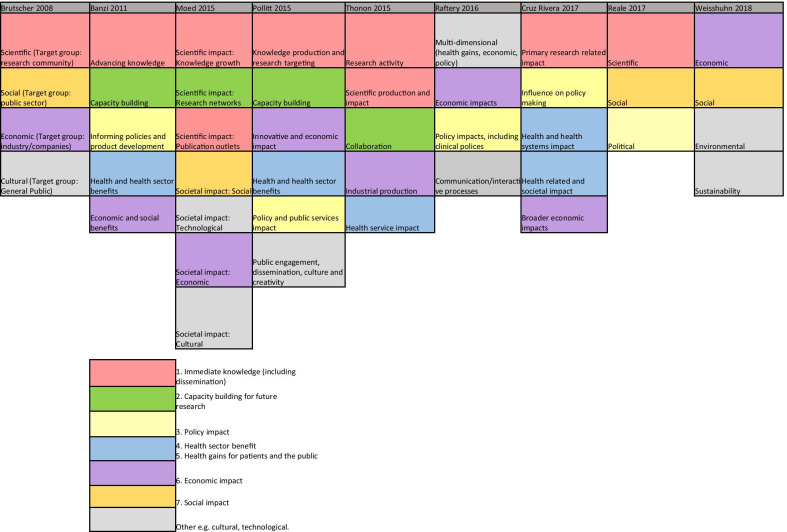
Categories of impact identified in the included literature reviews

i. Categorisation of impact for the purpose of impact assessment
  Nine reviews attempted to categorise the type of research impact being assessed according to who or what is affected by research, and how they are affected. In Fig. 2, colour coding was used to identify overlap between impact types identified in these reviews to produce a summary list of seven main impact categories.
  The first two categories of impact refer to the immediate knowledge produced from research and the contribution research makes to driving innovation and building capacity for future activities within research institutions. The former is often referred to as the academic impact of research. The academic impact of cancer research may include the knowledge gained from conducting experiments or performing clinical trials that is subsequently disseminated via journal publications. The latter may refer to securing future funding for cancer research, providing knowledge that allows development of later phase clinical trials or training cancer researchers of the future.
  The third category identified was the impact of research on policy. Three of the review articles included in this overview specifically focused policy impact evaluation [30–32]. In their review, Hanney et al. [30] suggested that policy impact (of health research) falls into one of three sub-categories: impact on national health policies from the government, impact on clinical guidelines from professional bodies, and impact on local health service policies. Cruz Rivera and colleagues [33] specifically distinguished impact on policy making from impact on clinical guidelines, which they described under health impact. This shows that the lines between categories will often blur.
  Impact on health was the next category identified and several of the reviews differentiated health sector impact from impact on health gains. For cancer research, both types of health impact will be important given that it is a health condition which is a major burden for healthcare systems and the patients they treat. Economic impact of research was the fifth category. For cancer research, there is likely to be close overlap between healthcare system and economic impacts because of the high cost of cancer care for healthcare services globally.
  In their 2004 article, Buxton et al. [34] searched the literature for examples of the evaluation of economic return on investment in health research and found four main approaches, which were referenced in several later reviews [19, 25, 35, 36]. These were (i) measuring direct cost savings to the health-care system, (ii) estimating benefits to the economy from a healthy workforce, (iii) evaluating benefits to the economy from commercial development and, (iv) measuring the intrinsic value to society of the health gain from research. In a later review [25], they added an additional approach of estimating the spill over contribution of research to the Gross Domestic Product (GDP) of a nation.
  The final category was social impact. This term was commonly used in a specific sense to refer to research improving human rights, well-being, employment, education and social inclusion [33, 37]. Two of the reviews which included this category focused on the impact of non-health related research (social sciences and agriculture), indicating that this type of impact may be less relevant or less obvious for health related disciplines such as oncology. Social impact is distinct from the term societal impact, which was used in a wider sense to describe impact that is external to traditional academic benefits [38, 39]. Other categories of impact identified that did not show significant overlap between the reviews included cultural and technological impact. In two of the literature reviews [33, 40], the authors provided a list of indicators of impact within each of their categories. In the review by Thonon et al. [40], only one (1%) of these indicators was specific to evaluating the impact of cancer research.
ii. Methods for data collection and analysis
  In total, 36 (90%) reviews discussed methods to collect or analyse the data required to conduct an impact evaluation. The common methods described, and the  strengths and weaknesses of each approach, are shown in Additional file 2: Table S1. Many authors advocated using a mixture of methods and in particular, the triangulation of surveys, interviews (of researchers or research users), and documentary analysis [20, 30–32]. A large number of reviews cautioned against the use of quantitative metrics, such as bibliometrics, alone [29, 30, 41–48]. Concerns included that these metrics were often not designed to be comparable between research programmes [49], their use may incentivise researchers to focus on quantity rather than quality [42], and these metrics could be gamed and used in the wrong context to make decisions about researcher funding, employment and promotion [41, 43, 45].
  Several reviews explained that the methods for data collection and analysis chosen for impact evaluation depended on both the unit of research under analysis and the rationale for the impact analysis [23, 24, 26, 31, 36, 50, 51]. Specific to cancer research, the unit of analysis may be a single clinical trial or a programme of trials, research performed at a cancer centre or research funded by a specific institution or charity. The rationale for research impact assessment was categorised in multiple reviews under four headings (“the 4 As”): advocacy, accountability, analysis and allocation [19, 20, 23, 24, 30–33, 36, 46, 52, 53]. Finally, Boaz and colleagues found that there was a lack of information on the cost-effectiveness of research impact evaluation methods but suggested that pragmatic, but often cheaper approaches to evaluation, such as surveys, were least likely to give in depth insights into the processes through which research impact occurred [31].
iii. Using a framework within a research impact evaluation
  Applied to research impact evaluation, a framework provides a way of organising collected data, which encourages a more objective and structured evaluation than would be possible with an ad hoc analysis. In total, 27 (68%) reviews discussed the use of a framework in this context. Additional file 2: Table S2 lists the frameworks mentioned in three or more of the included reviews. The most frequently described framework was the Payback Framework, developed by Buxton and Hanney in 1996 [54], and many of the other frameworks identified reported that they were developed by adapting key elements of the Payback framework. None of the frameworks identified were specifically developed to assess the impact of cancer research, however several were specific to health research. The unit of cancer research being evaluated will dictate the most suitable framework to use in any evaluation. The unit of research most suited to each framework is outlined in Additional file 2: Table S2.

#### Additional findings from the included reviews

The challenges of research impact evaluation were commonly discussed in these reviews. Several mentioned that the time lag [24, 25, 33, 35, 38, 46, 50, 53, 55] between research completion and impact occurring should influence when an impact evaluation is carried out: too early and impact will not have occurred, too late and it is difficult to link impact to the research in question. This overlapped with the challenge of attributing impact to a particular piece of research [24, 26, 33–35, 37–39, 46, 50, 56]. Many authors argued that the ability to show attribution was inversely related to the time since the research was carried out [24, 25, 31, 46, 53].

### Part II: Empirical examples of cancer research impact evaluation

#### Study characteristics

In total, 14 empirical impact evaluations relevant to cancer research were identified from the references lists of the literature reviews included in the first part of this study. These empirical studies were published between 1994–2015 by primary authors located in the UK (7/14; 50%), USA (2/14; 14%), Italy (2/14; 14%), Canada (2/14; 14%) and Brazil (1/14; 14%). Table 2 lists these studies with the rationale for each assessment (defined using the “4As”), the unit of analysis of cancer research evaluated and the main findings from each evaluation. The categories of impact evaluated, methods of data collection and analysis, and impact frameworks utilised are also summarised in Table 2 and discussed in more detail below.

**Table 2:** Examples of primary studies assessing the impact of cancer research

Author(s)	Title	Year	Location	Description	Unit of analysis	Main reason for assessment	Categories assessed	Methods used	Framework	Main findings	Strengths	Limitations
Brown ML, Nayfield SG, Shibley LM	Adjuvant therapy for stage III colon cancer: economic returns to research and cost-effectiveness of treatment	1994	USA	Evaluation of cost-effectiveness of adjuvant treatment for colorectal cancer and assessment of the return on investment to conduct clinical trial research	Cancer clinical trial	Accountability	Based on principles of economic evaluation. Economy, health	Cost effectiveness analysis (CEA) followed by evaluation of the social return on research investment. Human capital approach used to value return on investment	Nil	Cost effectiveness of treatment estimated at $2094 US dollars per year of life saved. The net present value of the return on the $10.84 million investment in the trial estimated at $1.66 billion dollars	The cost of conducting research incorporated into analysis of the value of the research investment. Time-period for assessment of the costs and benefits extended to 2020 to capture downstream effects	Assumptions made about adoption of trial results into practice rather than an assessment of actual practice change
Ugolini D, Bogliolo A, Parodi S, Casilli C, Santi L	Assessing research productivity in an oncology research institute: the role of the documentation centre	1997	Italy	Estimation of the academic impact of departments within a cancer research institution	Cancer research centre	Allocation	Academic impact	Bibliometric assessment using an journal impact factor based metric	Nil	Most publications from the research centre that were identified and analysed scored highly (8–10/10) on the normalised journal impact factor score	An early (1997) attempt to evaluate the academic impact of a cancer institute	Metric, quantitative based approach only looking at a narrow interpretation of impact. Using the impact factor of a journal to assess the quality of individual research articles and for allocation of resources
Ugolini D, Casilli C, Mela GS	Assessing oncological productivity: Is one method sufficient?	2002	Italy	Estimation of the contribution to academic cancer publications from different European countries using bibliometric methods	European countries	Analysis	Academic impact	Bibliometric method using the number of occurrences of an oncological publication in a journal (by author country of origin) compared to country population/GDP and the "mean impact factor" of the occurrences of the publication by author	Nil	The UK made the highest contribution to European cancer publication output (21.12%), whereas Sweden performed best in the metric of number of publication occurrences versus country population and the Netherlands was ranked first for the mean impact factor of the occurrences	Comparison of two methods to understand how to assess the impact of cancer researchers' work	Quantitative metric used that does not assess the quality of the individual articles or the contribution of the authors to the work. The authors acknowledge the limitations of using bibliometrics alone
Coyle D, Grunfeld E, Wells G	The assessment of the economic return from controlled clinical trials	2003	Canada	Evaluation of the potential payback of conducting a clinical trial to test the optimal follow up for patients with colorectal cancer. Two trial designs were compared: an equivalence trial and an effectiveness trial (looking for a survival benefit from more intensive follow up)	Cancer clinical trial	Allocation	Economic and health impact (potential)	Assessment of “time to payback” for two alternative hypothetical cancer trials. This is the number of years until the returns from conducting a clinical trial outweigh the costs	Nil	An effectiveness clinical trial would be worthwhile to look for a 5% improvement in survival from more intensive follow up (if a 5% improvement is considered likely). An equivalence trial would not be a worthwhile investment	Ex-ante evaluation shows how impact assessment can prevent investment in cancer trials that are unlikely to be worthwhile	An evaluation of potential rather than realised impact. Any error in the assumptions made and results obtained in these types of analysis means that some potentially impactful trials will not be performed
Lewison G, Sullivan R	The impact of cancer research: how publications influence UK cancer clinical guidelines	2008	UK	Evaluation of the cancer research that are cited in UK clinical guidelines	Any type of cancer research as cited in guideline	Analysis	Policy/guidelines	Identification of 43 UK guidelines from three guideline series (NICE, SIGN and Clinical Evidence) and bibliometric software used to analyse the guideline citations. Comparison of the citations versus those in the world oncology literature over 3-year period	Nil	UK papers were cited more frequently in cancer clinical guidelines than expected from their presence in the world oncology literature. The publications were generally more clinical than basic	Outlines and executes a method for evaluating the impact of cancer research on guidelines	The authors highlight that small clinical trials with negative outcomes are unlikely to be cited in guidelines, and discuss the limitations of using clinical guideline impact as a surrogate for practice change
Lewison G, Tootell S, Roe P, Sullivan R	How do the media report cancer research? A study of the UK's BBC website	2008	UK	Evaluation of the impact of cancer research on the BBC news website (accessed by 13.2 million people annually in the UK (2008 figures))	Any cancer research as cited in the media	Analysis	Media impact (dissemination)	Search of BBC archive health Section 1998–2006. Percentage of BBC stories focusing on different cancer sites were compared with the UK's burden of disease (WHO 2002). Research level (basic versus clinical) of cited papers was determined. The potential citation rate (PCI), actual citation rate (ACI) and funding of any cited paper compared to the global oncology research papers	Nil	Research on breast, cervical and skin cancer are over-reported in the media compared to their burden of disease whereas lung cancer is under-reported. New and improved drugs are the research topic most cited. UK research was over-cited in the UK media compared to its place in world oncology research	Novel attempt to investigate the impact of research in the media. Methodology clearly explained	Only one media website archive used for analysis
Saad et al.	The geography of clinical cancer research: analysis of abstracts presented at the American Society of Clinical Oncology Annual Meetings	2009	Brazil	Evaluation of cancer research disseminated at an international conference	Individual research projects presented in abstract form at a conference	Analysis	Academic impact and dissemination	Bibliometric analysis of a sample of abstracts		Over 50% of abstracts were from the USA. Clinical trials were more likely than "other" types of research to be presented in poster or oral form (vs publication only)	Large, international cancer conference and several years of submissions chosen for the analysis	Analysed only a sample (10%) of all abstracts, no comparison between the two time periods sampled performed and no information on the abstracts that were submitted but rejected
Lewison G, Markusova V	The evaluation of Russian cancer research	2010	UK	Evaluation of the extent of cancer research in Russia, if research meets the needs of the country, and how the impact of research compares on a world scale	Cancer research performed by one country	Analysis	Academic/scholarly impact	Quantification of number of cancer publications compared to the national wealth and disease burden of cancer in Russia compared to other countries. Citation scores for Russian cancer publications compared with the citations to cancer papers worldwide in the same years. Analysis of collaboration on Russian cancer research papers	Nil	Russia publishes one sixth as many cancer papers as its wealth and disease burden would suggest. Clinical cancer research papers receive more citation than basic ones (the reverse of what is seen elsewhere). Russian cancer research scores well for the percentage of reviews and collaboration in cancer research is dominated by former socialist states. Russian cancer research is incorporated into UK clinical guidelines, but rarely into UK media	Multiple indicators used to map some important impacts of cancer research	Mainly quantitative indicators used. No assessment of broader aspects of societal impact. Only looked at the impact on UK clinical guidelines and media rather that in Russia (likely to be much higher)
Lakdawalla DN, Sun EC, Anupam BJ, Reyes CM, Goldman DP, Philipson TJ	An economic evaluation of the war on cancer	2010	USA	Evaluation of the returns on investment from the USA government into cancer research from 1971	National cancer research	Accountability	Economic impact from a societal perspective	Quantification of gains in cancer survival using willingness to pay estimates derived from the literature and a comparison with the cost in cancer spending	Nil	Cancer survival increased by 3.9 years from 1988–2000 which equates to 23 million additional life years. The authors estimate that an average life year is worth 82,000 dollars and therefore the value of this survival is monetised at 1.9 trillion dollars in social value. Uses an 18-year time lag from research investment to survival outcome attributable to that research	Attempts to monetise a complex concept of willingness to pay for cancer survival. Detailed description of analysis	Complex methods, willingness to pay values from previous literature. The actual investment costs in different cancer types and different research types (basic versus trials) not outlined
Montague S, Valentim R	Evaluation of RT&D: from "prescriptions for justifying" to "user-orientated guidance for learning"	2010	Canada	Evaluation of the impact of a cancer RCT (MA17), which reported that an aromatase inhibitor called letrozole reduced rates of disease recurrence when given to patients after radical treatment for breast cancer	One cancer trial	Analysis	Multiple types of impact (based on CAHS framework)	Canadian Academy of Health Sciences Framework (CAHS) used to guide impact evaluation. Indicators of impact from CAHS framework combined with Bennett’s hierarchy	CAHS model, Bennett hierarchy	Impacts in all categories of the CAHS can be identified for this trial. The authors produce an impact timeline to show the pathway to impact in chronological order	Use of a conceptual framework means that the impact of this cancer trial is communicated in a transparent and organised fashion. The use of the hierarchy of events helps to demonstrate the processes through which impact has occurred	The theory of action hierarchy makes assumptions that prior events influence events higher up the chain
Sullivan R, Lewison G, Purushotham AD	An analysis of research activity in major UK cancer centres	2011	UK	Evaluation of the impact of research from UK major cancer centres on knowledge production (citation impact), clinical management, and the general-public (via media citation)	Research from cancer centres in the UK	Analysis	Academic/scholarly, clinical management, general public/media	Academic papers linked with cancer centres in the UK (via location or author) identified via database review. The journals of the papers were categorised by research level (basic versus clinical). Potential citation impact (PCI) and actual citation impact (ACI) calculated for the included papers and any international collaboration on the papers documented. Calculation of the number of papers from UK cancer centres that are reported in UK guidelines and in the media	Nil	UK cancer centres are heterogeneous in terms of their overall research output and the type of research performed. Overall, there was more focus on basic/fundamental research being published by UK cancer centres compared to applied research. There is heterogeneity in the proportion of papers from UK cancer centres that are cited in guidelines and the media that does not correlate with the size of the centre or the conventional citation impact of the papers	The authors combine publication citation impact with other impacts (on guidelines and media) to give a broader overview of cancer centre research impact	Mainly quantitative measures of impact. The authors mention the potential future use of the Research Impact Framework but they do not use this or any other framework in their current analysis
Donovan C, Butler L, Butt AJ, Jones TH, Hanney SR	Evaluation of the impact of National Breast Cancer Foundation-funded research	2014	UK	Evaluation of the academic and wider impacts resulting from funding by a specific cancer charity in Australia (National Breast Cancer Foundation NBCF)	Programme of cancer research projects	Accountability	Multiple (based on Payback framework)	Desk analysis of data held by the charity, survey of chief investigators, 16 case studies, bibliometrics and international benchmarking using bibliometrics. The case studies used document and archival analysis, citation analysis, searching for citations in guidelines and interviews with principal investigators of projects	Payback	153 responses to a survey sent to 242 NBCF-funded researchers showed that the research they performed had impacts on drug development, higher degree attainment, capacity building for future research, policy, and health gain. Findings showed differences in impact between basic and applied cancer research, for example, basic research was more likely to lead to product development whereas applied research was more likely to impact on policy, behaviour, and practice	Transparent methodology using a recognised framework and a mixture of quantitative and qualitative methods to gain in depth insight into impact of the research	Impact from the perspective of the researchers, no other stakeholders approached to comment on perceived impact of the funded research
Glover M, Buxton M, Guthrie S, Hanney S, Pollitt A, Grant J	Estimating the returns to UK publicly funded cancer-related research in terms of the net value of improved health outcomes	2014	UK	Evaluation of the economic value of cancer research in the UK	National cancer research	Accountability	Economic	Economic analysis (bottom up approach used). Five main steps: Estimated spending on research between 1970–2009 (£15 billion), estimated the net monetary benefit using a monetized value for a QALY, estimated the cost of delivering the benefit by identifying a list of the most important interventions that had contributed to this benefit (via discussions with experts), estimated the proportion of NMB attributable to UK research and the time lapse between funding and health gain (using bibliometric analysis of clinical guidelines) and finally, the internal rate of return from cancer research on health benefits	Nil	Time lag between research spending and impact on health gain estimated as 15 years. Overall return on public spending on cancer research estimated as 10.1%	Considered the time lag between research spending and impact on health gain. Sensitivity analysis performed where possible and acknowledged areas of uncertainty. Acknowledged the purely quantitative nature of this assessment and accompanying case studies were performed (Guthrie et al.)	Multiple assumptions made to perform this economic analysis (recognised and outlined by the authors). The authors outline the difficulty in differentiating the impact of smoking cessation in their calculation of the impact of cancer research overall
Guthrie S, Pollitt A, Hanney S, Grant J	Investigating time lags and attribution in the translation of cancer research. A case study approach	2015	UK	Evaluation of the attribution of health gains due to investment in cancer research and the time lags between research investment and health gain being recognised using a case study approach	Six examples of cancer research topics which included clinical trials	Accountability	Health gain	Case study approach using mainly desk/documentary analysis	Nil	Individual narratives for each case study	The use of case studies enables the reader to understand the process through which impact has occurred and the time lines involved	Requires in depth documentary analysis to contextualise and explain case study specific impacts

#### Approaches to cancer research impact evaluation used in empirical studies

i. Categories of impact evaluated in cancer research impact assessments
  Several of the empirical studies focused on academic impact. For example, Ugolini and colleagues evaluated scholarly outputs from one cancer research centre in Italy [57] and in a second study looked at the academic impact of cancer research from European countries [58]. Saed et al. [59] used submissions to an international cancer conference (American Society of Clinical Oncology (ASCO)) to evaluate the dissemination of cancer research to the academic community, and Lewison and colleagues [60–63] assessed academic, as well as policy impact and dissemination of cancer research findings to the lay media.
  The category of the health impact was also commonly evaluated, with particular focus on the assessment of survival gains. Life years gained or deaths averted [64], life expectancy gains [65] and years of extra survival [66] were all used as indicators of the health impact attributable to cancer research. Glover and colleagues [67] used a measure of health utility, the quality adjusted life year (QALY), which combines both survival and quality of life assessments. Lakdawalla and colleagues [66] considered the impact of both research on cancer screening and treatments, and concluded that survival gains were 80% attributable to treatment improvement. In contrast, Glover and colleagues [67] acknowledged the importance of improved cancer therapies due to research but also highlight the major impacts from research around smoking cessation, as well as cervical and bowel cancer screening. Several of these studies that assessed health impact, also used the information on health gains to assess the economic impact of the same research [64–67].
  Finally, two studies [68, 69] performed multi-dimensional research impact assessments, which incorporated nearly all of the seven categories of impact identified from the previous literature (Fig. 2). In their assessment of the impact of research funded by one breast cancer charity in Australia, Donovan and colleagues [69] evaluated academic, capacity building, policy, health, and wider economic impacts. Montague and Valentim [68] assessed the impact of one randomised clinical trial (MA17) which investigated the use of a hormonal medication as an adjuvant treatment for patients with breast cancer. In their study, they assessed the dissemination of research findings, academic impact, capacity building for future trials and international collaborations, policy citation, and the health impact of decreased breast cancer recurrence attributable to the clinical trial.
ii. Methods of data collection and analysis for cancer research impact evaluation
  Methods for data collection and analysis used in these studies aligned with the categories of impact assessed. For example, studies assessing academic impact used traditional bibliometric searching of publication databases and associated metrics. Ugolini et al. [57] applied a normalised journal impact factor to publications from a cancer research centre as an indicator of the research quality and productivity from that centre. This analysis was adjusted for the number of employees within each department and the scores were used to apportion 20% of future research funding. The same bibliometric method of analysis was used in a second study by the same authors to compare and contrast national level, cancer research efforts across Europe [58]. They assessed the quantity and the mean impact factor of the journals for publications from each country and compared this to the location-specific population and GDP. A similar approach was used for the manual assessment of 10% of cancer research abstracts submitted to an international conference (ASCO) between 2001–2003 and 2006–2008 [59]. These authors examined if the location of authors affected the likelihood of the abstract being presented orally, as a face-to-face poster or online only.
  Lewison and colleagues, who performed four of the studies identified [60–63], used a different bibliometric method of publication citation count to analyse the dissemination, academic, and policy impact of cancer research. The authors also assigned a research level to publications to differentiate if the research was a basic science or clinical cancer study by coding the words in the title of each article or the journal in which the paper was published. The cancer research types assessed by these authors included cancer research at a national level for two different countries (UK and Russia) and research performed by cancer centres in the UK.
  To assess policy impact these authors extracted journal publications from cancer clinical guidelines and for media impact they looked at publications cited in articles stored within an online repository from a well-known UK media organisation (British Broadcasting Co-operation). Interestingly, most of the cancer research publications contained in guidelines and cited in the UK media were clinical studies whereas a much higher proportion published by UK cancer centres were basic science studies. These authors also identified that funders of cancer research played an critical role as commentators to explain the importance of the research in the lay media. The top ten most frequent commentators (commenting on > 19 media articles (out of 725) were all representatives from the UK charity CRUK.
  A combination of clinical trial findings and documentary analysis of large data repositories were used to estimate health system or health impact. In their study, Montague and Valentim [68] cited the effect size for a decrease in cancer recurrence from a clinical trial and implied the same health gains would be expected in real life for patients with breast cancer living in Canada. In their study of the impact of charitable and publicly funded cancer research in the UK, Glover et al. [67] used CRUK and Office for National Statistics (ONS) cancer incidence data, as well as national hospital databases listing episodes of radiotherapy delivered, number of cancer surgeries performed and systemic anti-cancer treatments prescribed, to evaluate changes in practice attributable to cancer research. In their USA perspective study, Lakdawalla et al. [66] used the population-based Surveillance, Epidemiology and End Results Program (SEER) database to evaluate the number of patients likely to be affected by the implementation of cancer research findings [66]. Survival calculations from clinical trials were also applied to population incidence estimates to predict the scale of survival gain attributable to cancer research [64, 66].
  The methods of data collection and analysis used for economic evaluations aligned with the categories of assessment identified by Buxton in their 2004 literature review [34]. For example, three studies [65–67] estimated direct healthcare cost savings from implementation of cancer research. This was particularly relevant in one ex-ante assessment of the potential impact of a clinical trial testing the equivalence of using less intensive follow up for patients following cancer surgery [65]. These authors assessed the number of years it would take (“years to payback”) of implementing the hypothetical clinical trial findings to outweigh the money spent developing and running the trial. The return on investment calculation was performed by estimating direct cost savings to the healthcare system by using less intensive follow up without any detriment to survival.
  The second of Buxton’s categories was an estimation of productivity loss using the human capital approach. In this method, the economic value of survival gains from cancer research are calculated by measuring the monetary contribution from patients surviving longer who are of working age. This approach was used in two studies [64, 66] and in both, estimates of average income (USA) were utilised. Buxton’s fourth category, an estimation of an individual’s willingness to pay for a statistical life, was used in two assessments [65, 66], and Glover and colleagues [67] adapted this method, placing a monetary value on the opportunity cost of QALYs forgone in the UK health service within a fixed budget [70]. One of the studies that used this method identified that there may be differences in how patients diagnosed with distinct cancer types value the impact of research on cancer specific survival [66]. In particular, individuals with pancreatic cancer seemed to be willing to spend up to 80% of their annual income for the extra survival attributable to implementation of cancer research findings, whereas this fell to below 50% for breast and colorectal cancer. Only one of the studies considered Buxton’s third category of benefits to the economy from commercial development [66]. These authors calculated the gain to commercial companies from sales of on-patent pharmaceuticals and concluded that economic gains to commercial producers were small relative to gains from research experienced by cancer patients.
  The cost estimates used in these impact evaluations came from documentary analysis, clinical trial publications, real-life data repositories, surveys, and population average income estimates. For example, in one study, cost information from NCI trials was supplemented by using a telephone phone survey to pharmacies, historical Medicare documents and estimates of the average income from the 1986 US Bureau of the Census Consumer Income [64]. In their study, Coyle et al. [65] costed annual follow up and treatment for cancer recurrence based on the Ontario Health Insurance plan, a cost model relevant to an Ottawa hospital and cost estimates from Statistics Canada [71]. The data used to calculate the cost of performing cancer research was usually from funding bodies and research institutions. For example, charity reports and Canadian research institution documents were used to estimate that it costs the National Cancer Institute in Canada $1500 per patient accrued to a clinical trial [65]. Government research investment outgoings were used to calculate that $300 billion was spent on cancer research in the USA from 1971 to 2000, 25% of which was contributed by the NCI [66] and that the NCI spent over $10 million USD in the 1980s to generate the knowledge that adjuvant chemotherapy was beneficial to colorectal cancer patients [64]. Charity and research institution spending reports, along with an estimation of the proportion of funds spent specifically on cancer research, were used to demonstrate £15 billion of UK charity and public money was spent on cancer research between 1970 and 2009 [67].
  Lastly, the two studies [68, 69] which adopted a multi-category approach to impact assessment used the highest number and broadest range of methods identified from the previous literature (Additional file 2: Table S1). The methods utilised included surveys and semi-structured telephone interviews with clinicians, documentary analysis of funding and project reports, case studies, content analysis of media release, peer review, bibiliometrics, budget analysis, large data repository review, and observations of meetings.
iii. Frameworks for cancer research impact evaluation
  Only two of the empirical studies identified used an impact framework. Unsurprisingly, these were also the studies that performed a multi-category assessment and used the broadest range of methods within their analyses. Donovan et al. [69] used the Payback framework (Additional file 2: Table S2) to guide the categories of impact assessed and the questions in their researcher surveys and interviews. They also reported the results of their evaluation using the same categories: from knowledge production, through capacity building to health and wider economic impacts. Montague and Valentim [68] used the Canadian Academy Health Services (CAHS) Framework (Additional file 2: Table S2). Rather than using the framework in it is original form, they arranged impact indicators from the CAHS framework within a hierarchy to illustrate impacts occurring over time. The authors distinguished short term, intermediate and longer-term changes resulting from one clinical cancer trial, aligning with the concept of categorising impacts based on when they occur, which was described in one of the literature reviews identified in the first part of this study [33].
  Lastly, the challenges of time lags and attribution of impact were identified and addressed by several of these empirical studies. Lewison and colleagues tracked the citation of over 3000 cancer publications in UK cancer clinical guidelines over time [61], and in their analysis Donovan et al. [69] explicitly acknowledged that the short time frame between their analysis and funding of the research projects under evaluations was likely to under-estimate the impact achieved. Glover et al. [67] used bibliometric analysis of citations in clinical cancer guidelines to estimate the average time from publication to clinical practice change (8 years). They added 7 years to account for the time between funding allocation and publication of research results giving an overall time lag from funding cancer research to impact of 15 years. The challenge of attribution was addressed in one study by using a time-line to describe impacts occurring at different time-points but linking back to the original research in question [68]. The difficultly of estimating time lags and attributing impact to cancer research were both specifically addressed in a companion study [72] to the one conducted by Glover and colleagues. In this study, instead of quantifying the return on cancer research investment, qualitative methods of assessment were used. This approach identified factors that enhanced and accelerated the process of impact occurring and helped to provide a narrative to link impacts to research.

## Discussion

This study has identified several examples of the evaluation of the impact of cancer research. These evaluations  were performed over three decades, and mostly assessed research performed in high-income countries. Justification for the approach to searching the literature used  in this study is given by looking at the titles of the articles identified. In only 14% (2/14) was the word “impact” included, suggesting that performing a search for empirical examples of cancer research impact evaluation using traditional publication databases would have been challenging. Furthermore, all the studies identified were included within reviews of approaches to research impact evaluation, which negated the subjective decision of whether the studies complied with a particular definition of research impact.

Characteristics of research that were specifically relevant to cancer studies can be identified from these impact assessments. Firstly, many of these evaluations acknowledged the contribution of both basic and applied studies to the body of cancer research, and several studies categorised research publications based on this distinction. Second, the strong focus on health impact and the expectation that cancer research will improve health was not surprising. The focus on survival in particular, especially in economic studies looking at the value of health gains, reflects the high mortality of cancer as a disease entity. This contrasts with similar evaluations of musculoskeletal or mental health research, which have focused on improvements in morbidity [73, 74]. Third, several studies highlighted the distinction between research looking at different aspects of the cancer care continuum; from screening, prevention and diagnosis, to treatment and end of life care. The division of cancer as a disease entity by the site of disease was also recognised. Studies that analysed the number of patients diagnosed with cancer, or population-level survival gains, often used site-specific cancer incidence and other studies evaluated research relating to only one type of cancer [64, 65, 68, 69]. Lastly, the empirical examples of cancer research impact identified in this study confirm the huge investment into cancer research that exists, and the desire by many research organisations and funders to quantify a rate of return on that investment. Most of these studies concluded that cancer research investment far exceeded expectations of the return on investment. Even using the simple measure of future research grants attracted by researchers funded by one cancer charity, the monetary value of these grants outweighed the initial investment [69].

There were limitations in the approaches to impact evaluation used in these studies which were recognised by reflecting on the findings from the broader literature. Several studies assessed academic impact in isolation, and studies using the journal impact factor or the location of authors on publications were limited in the information they provided. In particular, using the journal impact factor (JIF) to allocate funding research which was used in one study, is now outdated and controversial. The policy impact of cancer research was commonly evaluated by using clinical practice guidelines, but other policy types that could be used in impact assessment [30], such as national government reports or local guidelines, were rarely used. In addition, using cancer guidelines as a surrogate for clinical practice change and health service impact could have drawbacks. For example, guidelines can often be outdated, irrelevant or simply not used by cancer clinicians and in addition, local hospitals often have their own local clinical guidelines, which may take precedent over national documents. Furthermore, the other aspects of policy impact described in the broader literature [30], such as impact on policy agenda setting and implementation, were rarely assessed. There were also no specific examples of social, environmental or cultural impacts and very few of the studies mentioned wider economic benefits from cancer research, such as spin out companies and patents. It may be that these types of impact were less relevant to cancer research being assessed, however unexpected impacts may have be identified if they were considered at the time of impact evaluation.

Reflecting on how the methods of data collection and analysis used in these studies aligned with those listed in Additional file 2: Table S1 bibliometrics, alternative metrics (media citation), documentary analysis, surveys and economic approaches were often used. Methods less commonly adopted were interviews, using a scale and focus groups. This may have been due to the time and resource implications of using qualitative techniques and more in depth analysis, or a lack of awareness by authors regarding the types of scales that could be used. An example of a scale that could be used to assess the impact of research on policy is provided in one of the literature reviews identified [30]. The method of collecting expert testimony from researchers was utilised in the studies identified, but there were no obvious examples of testimony about the impact of cancer research from stakeholders such as cancer patients or their families.

Lastly, despite the large number of examples identified from the previous literature, a minority of the empirical assessments used an impact framework. The Payback Framework, and an iteration of the CAHS Framework were used with success and these studies are excellent examples of how frameworks can be used for cancer research impact evaluation in future. Other frameworks identified from the literature (Additional file 2: Table S2) that may be appropriate for the assessment of cancer research impact in future include Anthony Weiss’s logic model [75], the research impact framework [76] and the research utilisation ladder [77]. Weiss’s model is specific to medical research and encourages evaluation of how clinical trial publication results are implemented in practice and lead to health gain. He describes an efficacy-efficiency gap [75] between clinical decision makers becoming aware of research findings, changing their practice and this having impact on health. The Research Impact Framework, developed by the Department of Public Health and Policy at the UK London School of Hygiene and Tropical Medicine [76], is an aid for researchers to self-evaluate their research impact, and offers an extensive list of categories and indicators of research which could be applied to evaluating the impact of cancer research. Finally, Landry’s Research Utilisation Ladder [77] has similarities to the hierarchy used in the empirical study by Montegue and Valentim [68], and focuses on the role of the individual researcher in determining how research is utilised and its subsequent impact.

**Fig. 3:**
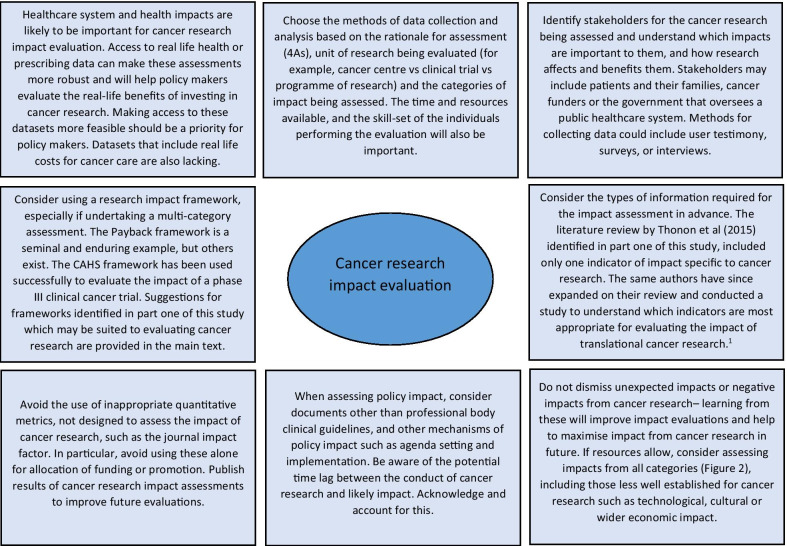
Suggestions for approaching cancer research impact evaluation. ^1^Thonon F, Boulkedid R, Teixeira M, Gottot S, Saghatchian M, Alberti C. Identifying potential indicators to measure the outcome of translational cancer research: a mixed methods approach. Health Res Policy Syst. 2015;13:72

Reflecting on the strengths and limitations of the empirical approaches to cancer research impact identified in this study, Fig. 3 outlines recommendations for the future. One of these recommendations refers to improving the use of real-life data to assess the actual impact of research on incidence, treatment, and outcomes, rather than predicting these impacts by using clinical trial results. Databases for cancer incidence, such as SEER (USA) and the Office of National Statistics (UK), are relatively well established. However, those that collect data on treatments delivered and patient outcomes are less so, and when they do exist, they have been difficult to establish and maintain and often have large quantities of missing data [78, 79]. In their study, Glover et al. [67] specifically identified the lack of good quality data documenting radiotherapy use in the UK in 2012.

The recommendations also suggest that impact assessment for cancer and other health research could be made more robust by giving researchers access to cost data linked to administrative datasets. This type of data was used in empirical impact assessments performed in the USA [64, 66] because the existing Medicare and Medicaid health service infrastructure collects and provides access to this data. In the UK, hospital cost data is collected for accounting purposes but this could be unlocked as a resource for research impact assessments going forward. A good example of where attempts are being made to link resource use to cost data for cancer care in the UK is through the UK Colorectal Cancer Intelligence Hub [80].

Lastly, several empirical examples highlighted that impact from cancer research can be increased when researchers or research organisations advocate, publicise and help to interpret research findings for a wider audience [60, 72]. In addition, it is clear from these studies that organisations that want to evaluate the impact of their cancer research must also appreciate that research impact evaluation is a multi-disciplinary effort, requiring the skills and input from individuals with different skill sets, such as basic scientists, clinicians, social scientists, health economists, statisticians, and information technology analysts. Furthermore, the users and benefactors from cancer research, such as patients and their families, should not be forgotten, and asking them which impacts from cancer research are important will help direct and improve future evaluations.

The strengths of this study are the broad, yet systematic approach used to identify existing reviews within the research impact literature. This allowed a more informed assessment of cancer research evaluations than would have been possible if a primary review of these empirical examples had been undertaken. Limitations of the study include the fact that the review protocol was not registered in advance and that one researcher screened the full articles for review. The later was partly mitigated by using pre-defined inclusion criteria.

## Conclusions

Impact assessment is a way of communicating to funders and patients the merits of undertaking cancer research and learning from previous research to develop better studies that will have positive impacts on society in the future. To the best of our knowledge, this is the first review to consider how to approach evaluation of the impact of cancer research. At the policy level, a lesson learned from this study for institutions, governments, and funders of cancer research, is that an exact prescription for how to conduct cancer research impact evaluation cannot be provided, but a multi-disciplinary approach and sufficient resources are required if a meaningful assessment can be achieved. The approach to impact evaluation used to assess cancer research will depend on the type of research being assessed, the unit of analysis, rationale for the assessment and the resources available. This study has added to an important dialogue for cancer researchers, funders and patients about how cancer research can be evaluated and ultimately how future cancer research impact can be improved.

## Supplementary information

**Additional file 1.** Research Council UK Impact definition, summary of search terms for part one, and inclusion criteria for both parts of the study.

**Additional file 2: Table S1** (List of methods for research impact evaluation) and **Table S2** (List if frameworks for research impact evaluation).

## Data Availability

Additional files included. No primary research data analysed.

## Abbreviations

NCI: National Cancer Institute
USA: United States of America
US: United States
UK: United Kingdom
CRUK: Cancer Research UK
MeSH: Medical subject heading
PRISMA: Preferred reporting items for systematic reviews and meta-analysis
GDP: Gross Domestic Product
ASCO: American Society of Clinical Oncology
SEER: Surveillance, Epidemiology and End Results Program
JIF: Journal impact factor
REF: Research evaluation framework
HTA: Health Technology Assessment
PhD: Doctor of Philosophy
RAND: Research and Development
QALY: Quality adjusted life year
CAHS: Canadian Academy of Health Sciences
ONS: Office for National Statistics
