# Supplementary material for: Evaluating cancer research impact: lessons and examples from existing reviews on approaches to research impact assessment
Source: Health Res Policy Syst. 2021 Mar 11;19:36. doi: 10.1186/s12961-020-00658-x (PMC7953786; doi:10.1186/s12961-020-00658-x)
Supplement: Supplementary file 1 — Additional file 1. Research Council UK Impact definition, summary of search terms for part one, and inclusion criteria for both parts of the study. [file 12961_2020_658_MOESM1_ESM.docx]

**Additional File 1**

**RCUK Research Impact definition**

***“Academic impact***
The demonstrable contribution that excellent research makes to academic advances, across and within disciplines, including significant advances in understanding, methods, theory and application.

***Economic and societal impacts***
The demonstrable contribution that excellent research makes to society and the economy. Economic and societal impacts embrace all the extremely diverse ways in which research-related knowledge and skills benefit individuals, organisations and nations.”

**Part I Inclusion and Exclusion Criteria**

**Inclusion criteria**

“Review” or “overview” mentioned in abstract or methods

Review of methods to assess or evaluate the impact of research

1998-2019

Can include part of a document as long as that is a standalone review e.g. a chapter in a PhD thesis or a literature review in a supplementary appendix

Articles reviewing the methodology of approaches to impact assessment were permitted, as well as reviews of empirical impact evaluations. Narrative, as well as systematic reviews were included because it was decided prior to conducting the study that both review types would contribute to understanding approaches to research impact evaluation and to identifying evaluations of cancer research impact. ([16](#_ENREF_16)) This was a purposefully broader approach compared to the methodology applied to conduct an “umbrella review” or “overview of reviews” ([17-19](#_ENREF_17)), which traditionally include only systematic reviews of the literature.

**Exclusion criteria**

Description of one framework e.g. REF/Payback with no review of approaches to existing approaches to research impact evaluation

Primary, empirical examples analysing research impact

PowerPoint® presentations, visual or multimedia

Editorials, blogs, short opinion pieces, workshops, books.

Instructions on how to use an impact assessment framework

Website URL with no identifiable document

Documents focusing on one country (Reviews of methods from several different countries to evaluate research impact *are* permitted)

Articles only focusing on the *definition* of research impact

Articles only focusing on “health impact assessment” (not the same as the impact of health research)

Articles describing the development of a new framework with a brief mention of a scoping review in the methodology are excluded.

**Part II Inclusion and Exclusion Criteria**

**Inclusion**

Empirical examples of research impact assessment.

Research under evaluation is specifically cancer research. This can include basic science or applied research within any cancer domain.

**Exclusion**

Primary evaluations of research impact when the research may include cancer research or clinical trials, but this is not the primary focus of the research or research programme being evaluated, are excluded.

**Search terms**

**Keywords:**

Impact (title)

AND (framework* OR pathway* OR tool* OR measur* OR categor* OR demonstr* OR evaluat* OR method* OR model* OR metric* OR assess*)

Research (title)

**MeSH terms:**

Research, Biomedical Research, Health Services Research.

**Search strategy Ovid Medline**

| 1 | impact.ti. |
| --- | --- |
| 2 | (framework* or pathway* or tool* or measur* or categor* or demonstr* or evaluat* or model* or method* or metric* or assess*).ti. |
| 3 | 1 and 2 |
| 4 | exp Research/ |
| 5 | exp Biomedical Research/ |
| 6 | exp Health Services Research/ |
| 7 | research.ti. |
| 8 | 4 or 5 or 6 or 7 |
| 9 | 3 and 8 |
| 10 | limit 9 to (english language and yr="1998 -Current") |
