# Supplementary material for: Evaluating cancer research impact: lessons and examples from existing reviews on approaches to research impact assessment
Source: Health Res Policy Syst. 2021 Mar 11;19:36. doi: 10.1186/s12961-020-00658-x (PMC7953786; doi:10.1186/s12961-020-00658-x)
Supplement: Supplementary file 2 — Additional file 2: Table S1 (List of methods for research impact evaluation) and Table S2 (List if frameworks for research impact evaluation). [file 12961_2020_658_MOESM2_ESM.docx]

Table S1: Methods of data collection for the purposes of research impact assessment.

| Method of data collection | Reference ID number (Table 1) | Explanation | Advantage | Disadvantage |
| --- | --- | --- | --- | --- |
| Desk/documentary analysis | 1,4,7,10,14,15,  16,18,19,22,25,  26,27,34,36,39,  40 | Umbrella terms referring to the use of documents and data to analyse research impact. See Hanney et al 2003 review Additional File 1: Elements of a protocol for documentary analysis. | Time efficient, likely to be cost-effective. Can be applied to a wide range of sources, e.g. publications, guidelines, conference proceedings, reports, and generate quantitative or qualitative findings. | No standard methodology for analysis. Relies on the quality of the primary document. |
| Questionnaires/Surveys | 1,4,5,7,8,14,15,  16,18,26,27,31,  34,36,39,40 | Includes online, telephone and paper/postal surveys. | Can be used for a wide range of stakeholders and disciplines, and often cost effective. Can identify areas to focus on in an interview. | Relies on robust response rate and access to respondents - concerns on the burden to answer them and the accuracy if incomplete data. Inherent bias between those who respond and those that do not respond. Hanney et al 2007 estimate that an average response rate is approximately two thirds. This raises questions about whether the respondents are representative of the population of interest. |
| Interviews | 1,4,5,7,10,15,16,  19,25,26,27,32,34,  35,36,39,40 | Can be structured, semi-structured or unstructured and open-ended. Can be conducted face-to-face, over the telephone or on a video link. (See Hanney et al 2003 Additional File 2: Draft interview schedule for assessing research utilisation in policy-making). | Can gain personal perspectives from individuals, for example researchers or users of research and allows in depth analysis. | Time consuming, interviewers need to be skilled so as not to introduce bias, may not be generalisable. |
| Case studies or impact narratives | 1,4,5,7,8,9,10,  15,16,18,19,25,26,  27,29,32,33,34,  35,39,40 | A narrative description of the impact of research. Other methods may have been used to collect data that is used within the impact narrative. | These narratives often take a wide perspective of research impact, can provide evidence of a pathway to and a context for the research impact described. | Time consuming and costly. Case study selection may be biased towards only positive or high achieving research examples. |
| Bibliometrics (including citation analysis) | 5,7,8,10,12, 13,15,17,18,19,  20,22,23,24,25,26,  27,28,29,33,34,  35,38,39,40 | Quantitative analysis of research activity, usually in the form of journal publications and citations. | Low cost and burden, can be used for different research disciplines. | Focus on outputs and not necessarily a measure of quality or impact. Can discriminate against researchers who have been active for less time. Not comparable between disciplines, for example, humanities versus medical sciences. Often citation analysis only considers citations in publication databases and does not include other data sources such as books or reports. Open to gaming. |
| Alternative metrics | 17,18,22,23,25,  26,28,29,33,38 | Quantitative analysis of research activity, usually based on non-academic, internet-related, social media or lay media citations. | Captures different routes of research dissemination compared to traditional bibliometrics. Can be analysed in an automated fashion with a high degree of accuracy. | Similar to bibliometrics. Mentions on social media or the lay new media may be more an indication of dissemination and influence than impact. Open to manipulation and spamming. |
| Peer review | 1,5,7,12,14,15,16,  19,23,25,29,31,33,  35,38 | Relies on advisory service by experts in the field to assess the impact of the research in question. Material can be reviewed by a group of peers (allows discussion) or individuals. | Credibility within the academic community, can offer expert feedback for future improvement, flexible, can be conducted at any time during an impact analysis. | Subjective, not transparent, may be costly, requires facilitation, can be slow. Although peers may be expert in their field, they may not be experts at assessing research impact - may benefit from getting research user input also. Impractical to assess a broad area of research given that peers are generally experts in one field. Time, cost, and reporting bias issues around how information is presented to peers for review. |
| Economic evaluation strategies | 2,4,5,6,8,9,10,  11,12,15,18,20,  25,26,31,36,39 | An assessment of how the benefit of research compare to the opportunity cost of investing the same funding elsewhere. An umbrella term that incorporates many methods, for example, cost-benefit analysis, cost-utility analysis, cost-effectiveness, proportion of GDP, social return on investment (SROI), intellectual Property register and commercial income generated, research funding. | Offers an estimation (often monetary) of the return on research investment which may be especially useful to funders. A single monetary figure for the return on a specific research project means that a comparison with the return on the benefits from other research projects may be possible. | Challenging to monetise broad impacts such as health and to account for all research costs and benefits. |
| Using a scale | 1 | Using a scale to assess the extent of research use within a certain category. For example, using a scale to report the level of research utilisation in policymaking. (See Hanney 2003: Additional File 3: Draft scales of the level of research utilisation in health policy-making). | Relatively easy to carry out and gives an indication of the extent of the impact. Research projects could be assessed using the same scale to give consistency of reporting. | The disadvantages of any quantitative metric. Problems with ensuring a fair comparison between research items could be decreased by using the same person or same team to score the research projects. |
| Benchmarking | 5,14, | The act of comparing metrics, usually bibliometrics, in order to compare impact from different research studies. | May be useful on an institutional level as a tool to encourage improved research productivity. | Used alone this may not be a measure of impact. The benchmarking output requires careful interpretation in context. |
| Workshop or focus group | 7,34,35,40 | Discussion amongst a group of people, usually facilitated by a researcher. | May be used as a tool to evaluate the impact of research on a group of stakeholders. | Time and cost intensive to run and analyse. |
| Literature review or meta-analysis | 7,16,26,35,36,40 | An overview of the literature. | Usually straightforward to perform. May be used to understand the impact of current research in the context of other evidence on the same topic. | Measure of knowledge production and academic impact only. |
| User or expert testimony | 7,8,16,18 | A statement from the user of the research or an expert in the field that describes the impact of the research from their perspective. | Straightforward to collect and demonstrates the impact of research directly from the stakeholder perspective. | One perspective only. Experts in the field may not be away of the impact of research. |

Table S2: Frameworks for research impact assessment mentioned in three or more of the included literature reviews.

| **Framework** | **Main level intended for evaluation** | **Description** |
| --- | --- | --- |
| **Payback Framework** | Various | Developed at Brunel University in 1990s to evaluate the impact of health services research. Modified logic model with seven stages (0-6) and 5 main categories of benefits from research. |
| **Societal Impact Assessments and other frameworks (E.g. SIAMPI Evaluating Research in Context)** | Initial case studies aimed at a centre/institution level of assessment. | Developed through a collaboration between the UK's Economic and Social Research Council and researchers in the Netherlands. The focus is on social impact and there is an assumption is that interactions between researchers and stakeholders are important. The aim of this framework is learning rather than accounting or judging impact. |
| **Monetary/economic framework** | Various | Any method that attempts to evaluate the opportunity costs of research and its outputs. |
| **Research Impact framework (RIF)** | Individual researcher or project | Developed researchers at the London School of Hygiene and Tropical Medicine for healthcare researchers. Includes four categories of impact with sub-categories and indicators within each area. Based initially on a review of the literature and other assessment exercises, for example, the Research Assessment Exercise (RAE) and developed by interviewing researchers and applying the categories to research projects at their research centre. |
| **UK Research Excellence Framework (REF) and Research Assessment Exercise (RAE)** | National frameworks to evaluate institutions | Introduced by the UK government in 2014, the next assessment will be in 2021 and based on work done in Australia (the Research Quality Framework). Developed by the UK government to allocate funding to HEIs (20% funding allocated dependent on demonstrating the impact of research in 2014). The RAE was the previous national assessment effort by the UK government used prior to the REF in which impact was not explicitly included. |
| **Canadian Academy of Health Sciences (CAHS)** | Various | Developed by a panel of experts to provide a framework for assessing Canadian healthcare research and based on the Payback Framework. |
| **Australian national frameworks Research Quality Framework (RQF), Excellence in Research Australia (ERA), Measurement of Research Impact and Assessment (MORIA)** | National | RQF was developed by a conservative Australian government in 2004 but never employed due to a change in government in 2007. The aim was to use it to assess the quality of publicly (taxpayer) funded research. It predominantly used case studies, peer review to assess impact, and was a major influencer of the current REF in the UK. Instead, the ERA was introduced, but with a stronger focus on quantitative metrics. First used in 2010, then 2012, 2015 and is being used again in 2018. MORIA was developed for use at the grant review stage. |
| **Weiss Logic model** | Various. Developed to assess medical research. | Developed to assess medical research by psychiatrist Anthony Weiss. It is a modified logic model. |
| **Netherlands Royal Academy of Arts and Sciences (SEP)** | National, institution or programme level. Uses self-evaluation and intermittent external review of the institution at set intervals. Overlap with the SIAMPI approach. | Used to assess research performance generally, not just the impact of research. There is overlap with ERiC. SEP is the national assessment process but there was initially no framework or specific methodology specified to carry out the assessment. The ERiC was used for this purpose. |
| **Lavis exchange model** | Various | A conceptual model describing producer push, user pull and the exchange model. |
| **Research Utilisation ladder** | Various. Could be used at the project or individual researcher level. | Focuses on the role of the researcher in creating impact from their research. The ladder consists of six stages from the transmission of research results to those results being used by others in a different context to that of the original research. |
| **HTA Quebec model (CETS: Conseil d'evaluation des technologies)** | Programme of HTA research. | Developed to assess the impact of research for the HTA programme in Quebec. Uses case studies, documentary analysis, and interviews. |
| **Banzi** | Various. | This approach to impact evaluation is based on their 2011 umbrella literature review. |
| **Balanced scorecard** | Various. | A performance management tool that is not specific to research impact. Focuses on financial dimensions, customer, business process, learning, and growth. |
| **Canadian Institutes of Health Research framework (CIHR)** | Various. | Developed in 2005 by Canadian and international experts and based on the Payback framework. Pre-dated and provided a basis for the CAHS model. |
| **Program Assessment Rating Tool (PART)** | Research programme level. | This approach was developed to evaluate the research performance of all USA government research programmes during the time of the Bush administration (2003). It uses a survey format and focuses on impacts and efficiency. |
